# Influence of Intravenous Norepinephrine Administration on Melatonin Plasma Concentration in Awake and Anaesthetised Healthy Adult Volunteers

**DOI:** 10.1111/jpi.70188

**Published:** 2026-09-30

**Authors:** Marko MIrza Sahinovic, Ilonka Nadia de Keijzer, Manon Stern, Karin Marianne Vermeulen, Michel Maria Roger Fernand Struys, Anthony Ray Absalom, Johannes Pieter van den Berg, Jaap Jan Vos

**Affiliations:** ^1^ Department of Anaesthesiology University Medical Centre Groningen, University of Groningen Groningen The Netherlands; ^2^ Department of Epidemiology University Medical Centre Groningen, University of Groningen Groningen The Netherlands; ^3^ Department of Basic and Applied Medical Sciences Ghent University Gent Belgium

**Keywords:** circadian rhythm, delirium, general anaesthesia, melatonin, norepinephrine, perioperative care, pineal gland

## Abstract

Melatonin (MEL) is an endogenous hormone that regulates circadian rhythm, immune function and oxidative stress responses. Norepinephrine (NE), an endogenous catecholamine, increases blood pressure through the sympathetic nervous system and is commonly administered intraoperatively or in the clinically ill patient for hemodynamic support. Although endogenous NE is known to stimulate MEL synthesis, the effects of exogenous intravenous NE on plasma MEL concentration, particularly under general anaesthesia (GA), remain poorly characterised. This study examined the influence of intravenous NE on plasma MEL concentrations in healthy volunteers, both awake and under general anaesthesia. A secondary analysis was conducted using data from a prospective, single‐centre, cross‐over study involving volunteers who received stepwise NE infusions while awake and under total intravenous general anaesthesia. Arterial blood samples were collected to determine plasma MEL and NE concentrations. Linear mixed‐effects modelling was used to assess the relationship between NE and MEL concentrations. A total of 36 healthy volunteers were enroled, yielding 417 MEL‐NE samples. Intravenous NE administration was significantly associated with higher plasma MEL concentration, corresponding to an estimated 26% increase in MEL per 1 standard‐deviation increase in NE concentration (Estimate = 0.230, *p* < 0.001). MEL concentrations were approximately 43% higher in the awake phase compared to GA (Estimate = 0.356, *p* < 0.001). GA did not significantly modify the NE‐MEL association (Estimate = −0.02877, *p* = 0.528). Substantial positive correlation was observed between baseline MEL concentrations and the magnitude of the response to NE administration (*r* = 0.74). In conclusion, intravenous NE increases plasma MEL concentrations in both awake and anaesthetised individuals. Substantial interindividual variation in MEL response suggests underlying physiological differences. Future studies should explore the long‐term effects of NE administration on MEL concentrations.

**Trial Registration:** The study is registered in the Dutch Trial Register (NL9312).

## 1 Introduction

Melatonin (MEL) plays an important role in the regulation of circadian timing and neuroendocrine homoeostasis. It is secreted by the pineal gland during darkness, under control of a light‐sensitive pathway that begins in the retina and ends in the suprachiasmatic nucleus [1]. This pathway activates a multi‐synaptic circuit, culminating in norepinephrine (NE) release at the pineal gland, where it stimulates β_2_‐adrenergic receptors on pinealocytes, and promotes enzymatic conversion of serotonin to MEL [2]. Physiologically, melatonin secretion is primarily driven by synaptic NE release from postganglionic sympathetic fibres. As the pineal gland lies outside the blood–brain barrier, circulating NE may influence MEL synthesis.

During the perioperative period, patients experience profound physiological disturbances induced by surgical stress and anaesthetic drugs. In addition to well‐recognised cardiovascular and respiratory effects, these factors impair cognition [3, 4], disrupt circadian rhythms [5, 6, 7], and alter neuroendocrine and immune function [8, 9] contributing to postoperative morbidity and delayed recovery. Similar disturbances occur in critically ill and sedated patients in the intensive care setting. Given melatonin's key role in regulating circadian rhythm and modulating immune responses, altered MEL production during these states may contribute to both postoperative and critical illness‐related complications [10, 11].

General anaesthesia, most often administered during daytime, suppresses MEL secretion through pharmacological effects, despite the absence of light exposure due to closed and covered eyes [11]. Concurrently, many anaesthetic drugs lower blood pressure through vasodilatation and myocardial depression, necessitating administration of vasopressors to restore hemodynamic stability. Among these agents, NE is one of the most commonly used vasopressors, routinely administered in supraphysiological concentrations during anaesthesia and critical care to treat hypotension and maintain organ perfusion [12]. Although endogenous NE is known to stimulate MEL synthesis, the impact of exogenous intravenous NE administration on circulating MEL concentration in humans has not been systematically investigated.

The aim of this study was therefore twofold: (1) to describe the effects of exogenous intravenous administration of NE on MEL plasma concentrations in healthy volunteers and (2) to determine whether general anaesthesia modulates the NE–MEL interaction.

## 2 Material and Methods

### 2.1 Study Design and Participants

This was a pre‐planned secondary analysis of data from a prospective, single‐centre, cross‐over study involving 36 healthy volunteers. The trial was approved by the BEBO (Stichting Beoordeling Ethiek Biomedisch Onderzoek) Medical Ethics Committee (METc Assen; NL76998.056.21) and registered in the Dutch Trial Register (NL9312). Written informed consent was obtained from all participants prior to enrolment, and the study was conducted in accordance with the principles outlined in the Declaration of Helsinki.

The primary focus of the original study was to investigate the pharmacology of exogenous NE with and without concurrent administration of propofol and remifentanil. An equal number of male and female volunteers were recruited, stratified into three age groups: 18–34, 35–50 and 51–70 years. Exclusion criteria were pregnancy, cardiovascular disease, anticipated difficult ventilation or intubation, gastric or pulmonary disease that could interfere with study procedure, end‐stage kidney or liver failure, the use of drugs that interfere with synthesis, reuptake or metabolism of catecholamines (e.g., MAO inhibitors or antidepressant drugs). A detailed description of the methodology of the original study has been previously published [13, 14].

### 2.2 Study Interventions

The study was conducted in a quiet, secluded windowless preoperative ‘holding area’ of the operating theatre complex with a non‐dimmable lighting always on, in the presence of a certified anaesthetist responsible for the care and safety of the volunteers, an anaesthetic nurse and a research physician who ensured adherence to the study protocol. The ambient light intensity in the study area was approximately 200 lux.

Upon arrival, standard anaesthetic monitoring was applied, including electrocardiography (ECG), pulse oximetry (SpO_2_) and intermittent non‐invasive blood pressure monitor and processed electroencephalography (pEEG) monitoring using a Bispectral Index (BIS) monitor (Medtronic, Ireland). An intravenous (IV) cannula was inserted into a vein on the dorsum of the dominant hand and Ringer's lactate solution was administered at a flow rate of 10 mL.h^−1^ to maintain cannula patency. This cannula was exclusively used for drug administration. An intra‐arterial cannula was then inserted into a radial artery under local anaesthesia to allow beat‐to‐beat blood pressure measurements and arterial blood sampling.

Each volunteer completed two sequential study phases. In Phase 1, procedures were performed in the awake state. In Phase 2, the protocol was repeated under general anaesthesia induced and maintained with propofol and remifentanil. Study procedures took approximately 6 h to complete, with Phase 1 starting at approximately 09:00 in the morning, and Phase 2 starting at approximately 12:30.

### 2.3 Drug Administration Schemes and Blood Sampling

*In Phase 1*, a continuous infusion of NE (norepinephrine tartrate, CF, CentraFarm, Breda, The Netherlands) 20 µg mL^−1^ was initiated at 0.04 µg kg^−1^ min^−1^. The infusion rate was increased stepwise in four 15 min intervals by 0.04 µg kg^−1^ min^−1^ to a maximum rate of 0.20 µg kg^−1^ min^−1^. Following the final step, the infusion was discontinued, and a 30‐min wash‐out period was observed.

MEL and NE arterial blood samples were obtained before the start of the NE infusion, at the end of the second (0.08 µg kg^−1^ min^−1^), fourth (0.16 µg kg^−1^ min^−1^) and final (0.20 µg kg^−1^ min^−1^) infusion steps, and after the wash‐out phase. A total of five MEL samples per volunteer were intended to be collected during the awake phase (Figure 1).

**Figure 1:**
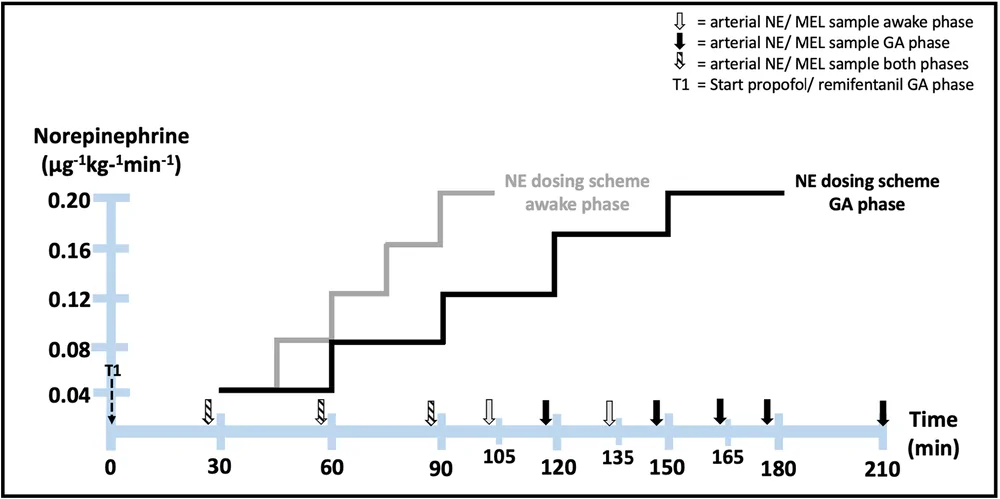
Study proceedings awake and GA phase.

*In Phase 2*, about 1 h after the completion of the washout phase and return of blood pressure to baseline values, volunteers were anaesthetised using propofol and remifentanil. After loss of response to verbal command, a laryngeal mask airway was inserted, and mechanical ventilation was initiated to maintain normocapnia and normoxia.

Fifteen minutes after the start of the propofol and remifentanil infusions, once a pseudo‐steady state of both drugs were presumed, NE administration was initiated to the same stepwise protocol as in Phase 1. If hypotension occurred (mean arterial pressure < 50 mmHg), NE administration was started earlier, at an appropriate infusion rate to ensure volunteer safety.

Fifteen minutes after the start of each infusion step, an electrical stimulus (50 mA, 100 Hz, EZstim III peripheral nerve stimulator, Halyard, Zaventem, Belgium) was applied to the medial malleolus for 30 s, to simulate a noxious surgical stimulus. After the final infusion step, a 30‐min wash‐out period was again observed. Arterial MEL and NE blood samples were collected before the start of the NE infusion, at the end of each infusion step, and at the end of the wash‐out period. An additional MEL and NE sample was collected halfway through the final infusion step (0.20 µg kg^−1^ min^−1^). In total, eight MEL/NE sample pairs were intended to be collected per volunteer during the general anaesthesia phase (Figure 1).

Upon completion of the washout phase, anaesthetic drug administration was discontinued. Volunteers were allowed to emerge from anaesthesia. After regaining responsiveness to verbal commands, volunteers were monitored in a recovery room until fully recovered and deemed ready for discharge.

### 2.4 Quantification of Plasma Melatonin and Norepinephrine Concentrations

Plasma MEL was measured using isotope dilution LC–MS/MS, a laboratory‐developed test validated under ISO 15189. The analytical method has been described previously by van Faassen et al. [15]. Sample preparation involved protein precipitation and solid‐phase extraction, with multiple reaction monitoring for quantification using stable isotope‐labelled internal standards. Calibration was performed with serial dilutions of reference standards. Melatonin concentrations are reported in pmol.L^−1^. The lower limit of detection (LLD) for MEL was 8 pmol.L^−1^. The lower limit of quantification (LLOQ) was 10 pmol.L^−1^. Intra‐assay coefficients of variation (CVs) were 8.9%, 3.9%, and 3.5% at melatonin concentrations of 20.5, 124 and 318 pmol.L^−1^, respectively. Corresponding inter‐assay CVs were 9.5%, 4.1% and 4.2% at concentrations of 21.5, 122 and 300 pmol.L^−1^, respectively.

NE was quantified by HPLC with electrochemical detection. Plasma was deproteinised with perchloric acid, followed by chromatographic separation on a reverse‐phase column. Internal quality controls ensured assay reliability.

All measurements were performed in duplicate, with intra‐ and inter‐assay CVs monitored for reproducibility.

### 2.5 Statistical Analysis

Descriptive statistics were used to summarise the data, and individual trajectories were visualised. The distribution of the outcome variables was assessed to check for normality and potential deviations using the Shapiro–Wilk test.

#### 2.5.1 Linear Mixed Effects Model Development

The influence of NE and GA on MEL plasma concentrations was analysed using a linear mixed‐effects model. A random intercept was included to account for interindividual variability in baseline MEL concentration. Initial model assessments showed non‐linearity and heteroscedasticity in MEL concentration data, necessitating log‐transformation of MEL concentrations to improve model fit.

A random slope for NE was introduced to assess interindividual variability in the response to NE. This resulted in numerical instability, necessitating standardisation (centring and scaling to one standard deviation) of the NE variable, which subsequently improved model stability.

GA was incorporated as a fixed effect to assess its association with MEL concentration. An interaction term between NE and GA was added to examine whether anaesthetic drugs modified the NE–MEL relationship; however, this term was not retained in the final model due to lack of improvement in model fit.

The final model included the scaled NE variable, GA status, and both a random intercept and slope for NE by subject.

#### 2.5.2 Model Diagnostics and Validation

Model assumptions were assessed through visual inspection of diagnostic plots. Residuals versus Fitted plots were used to evaluate linearity and homoscedasticity. Normal *Q*–*Q* plots were used to assess the normality of residuals, particularly focusing on alignment in the central quantiles with only minor deviations at the ends. Scale‐location plots were used to examine homogeneity of variance. Cook's Distance plots were used to detect influential outliers, ensuring that no single data point disproportionately influenced the model's estimates.

The overall model fit was evaluated using the Restricted Maximum Likelihood (REML) and AIC criterion.

#### 2.5.3 Confidence Intervals Calculation

Confidence intervals for fixed effects were computed using Wald‐type intervals derived from the standard errors of the estimates. For the random effects, confidence intervals for the standard deviations were calculated using the profile likelihood method. These intervals were then squared to obtain confidence intervals for the corresponding variances. All analyses were conducted using R software (version 4.4.1; R Foundation for Statistical Computing, Vienna, Austria).

## 3 Results

### 3.1 Patient Characteristics and Data Description

A total of 36 healthy volunteers were enroled in the study. One volunteer was excluded and replaced because the intravenous catheter migrated into the subcutaneous tissue during the procedure, without further sequelae [13]. Demographic data are presented in Table 1.

**Table 1:** Demographic data/sample‐related data/sample concentrations.

Demographics	Total (*n* = 36) median (IQR)	
Age (years)	39 (28–54)	
Sex (*n*, male)	18 (50%)	
Weight (kg)	70 (65.5–76.5)	
Height (cm)	173 (167–182)	
LBM	50.7 (48.3–60.3)	
*Samples*		
*N* samples max	468 (13 × 36)	
*N* samples collected	417	
Below LLD:		
*N* Samples	129	
PTNT all samples < LLD	4 (46 samples)	

Study phase and norepinephrine infusion step (µg kg^−1^ min^−1^).	Norepinephrine median (IQR) (nmol L^−1^)	Melatonin median (IQR) (pmol.L^−1^)
1. Awake 0	1.48 (1.25–1.88)	13.7 (8.81–24.3)
2. Awake 0.08	17.8 (14.0–19.9)	16.5 (8.41–26.6)
3. Awake 0.16	32.3 (28.7–35.4)	29.1 (10.4–43.1)
4. Awake 0.20	41.1 (37.6–44.8)	31.7 (12.1–57.2)
5. Awake Washout	2.23 (1.90–2.50)	12.4 (4.0–26.9)
6. GA 0	12.8 (10.1–19.9)	9.14 (4.0–15.3)
7. GA 0.04	10.4 (9.61–11.3)	4.0 (4.0–10.3)
8. GA 0.08	20.6 (17.8–22.5)	8.72 (4.0–16.1)
9. GA 0.12	29.3 (26.8–31.6)	9.85 (4.0–26.1)
10. GA 0.16	38.6 (33.5–43.0)	11.3 (4.0–37.6)
11. GA 0.20 (Halfway)	51.9 (45.5–55.1)	23.8 (4.0–55.4)
12. GA 0.20	49.6 (42.2–51.4)	12.0 (4.0–53.5)
13. GA Washout	2.54 (1.69–3.61)	12.2 (4.0–31.1)

Abbreviation: LBM = lean body mass.

The median and interquartile range (IQR) of the MEL and NE plasma concentrations are summarised in Table 1. A plot of individual MEL concentrations and boxplots of MEL concentrations per study phase can be seen in Figure 2.

**Figure 2:**
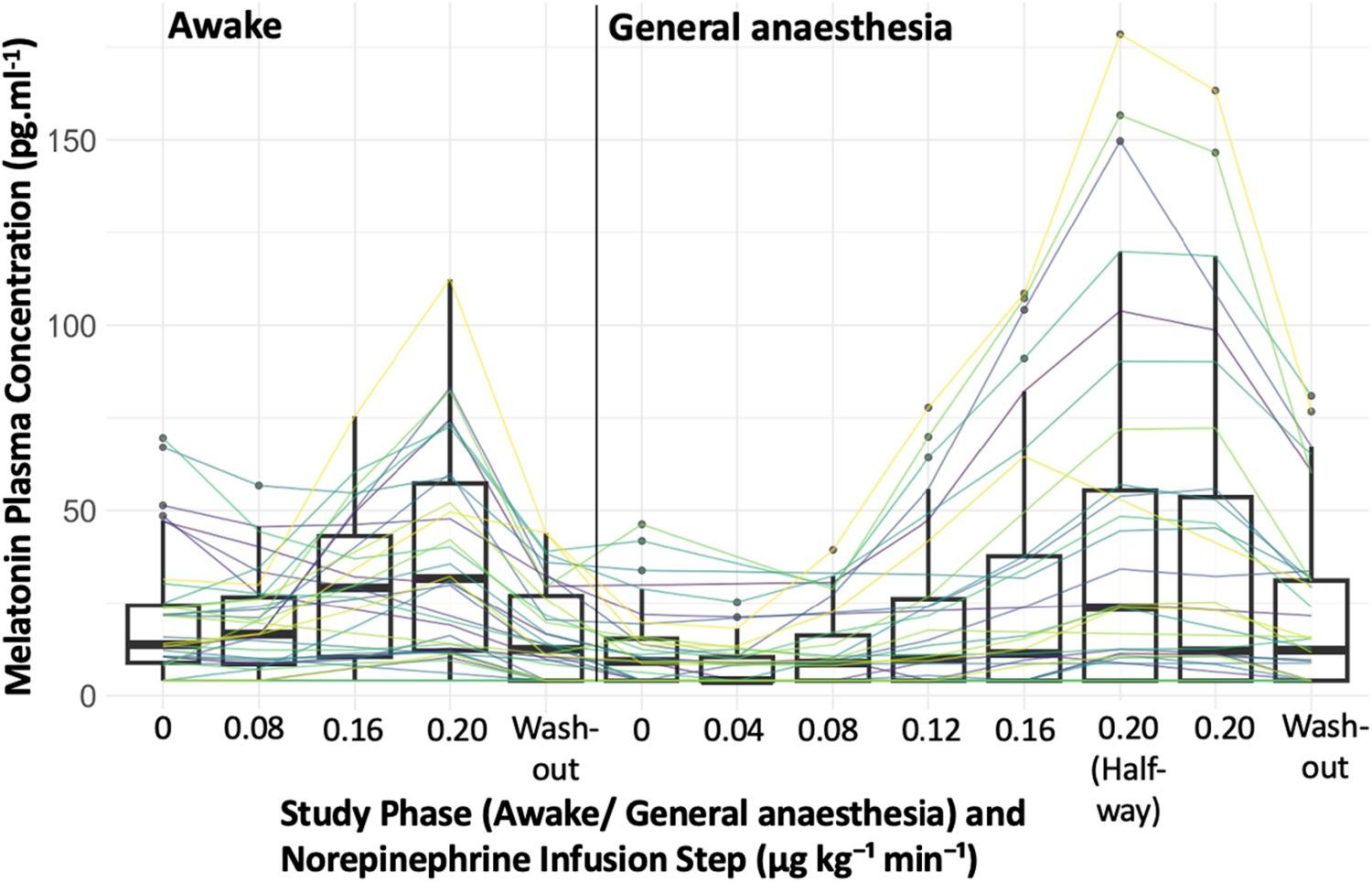
Plasma melatonin blood concentration. The *y*‐axis represents melatonin plasma concentration (pmol.L^−1^) and the *x*‐axis denotes the study phase and the norepinephrine infusion step (µg kg^−1^ min^−1^).

### 3.2 Available Data

A total of 417 paired MEL–NE samples were collected and analysed. In total, 51 samples were unavailable for analysis, primarily due to protocol deviations. These deviations occurred when NE administration was initiated earlier than planned to maintain blood pressure and ensure volunteer safety during the general anaesthesia phase.

At baseline, 25 volunteers had MEL concentrations > 10 pmol.L^−1^, while 11 volunteers had concentrations < 10 pmol.L^−1^. In four volunteers, all MEL measurements were below the lower limit of detection (LLD 8 pmol.L^−1^). Among the remaining volunteers, an additional 83 samples had MEL concentrations below the LLD resulting in a total of 129 samples with MEL concentrations below the LLD. To retain these data in the analyses, we assigned them each a value of 4 pmol.L^−1^.

### 3.3 Linear Mixed Effects Modelling

A linear mixed‐effects model was used to assess the relationship between MEL plasma concentration and several predictors, including NE plasma concentration and anaesthesia type (Table 2).

**Table 2:** Model 1. Linear mixed model fit by REML.

Random effects:					
Groups	Name	Variance	Std. dev.	CI 2.5%–97.5%	Corr
Ptnt_nr	(Intercept)	0.60847	0.78	0.38–0.98	
	Noradrenaline_scaled	0.04241	0.2059	0.02–0.078	0.74
Residual		0.18263	0.4274		

Fixed effects:							
	Estimate	Std. error	CI 2.5%–97.5%	df	*t* value	Pr(>|t|)	
(Intercept)	2.58328	0.13309	2.32–2.84	36.442	19.409	< 2.00E − 16	***
Noradrenaline_scaled	0.23048	0.04198	0.15–0.31	37.037	5.491	3.06E − 06	***
AnaesthesiaTypeAwake	0.35631	0.04449	0.27–0.44	351.03	8.009	1.72E − 14	***

*Note: t*‐tests use Satterthwaite's method [lmerModLmerTest]. Formula: log_melatonin ~ Noradrenaline_scaled + AnaesthesiaType + (1 + Noradrenaline_scaled | Ptnt_nr). REML criterion at convergence: 641.1. AIC(model_log_mixed_anaesthesia): 655.0799.

Signif. codes: 0 ‘***’ 0.001.

#### 3.3.1 Fixed Effects

A significant positive association was observed between scaled NE plasma concentration and log‐transformed MEL plasma concentration (Estimate = 0.230, CI = 0.15–0.31, *p* < 0.001), indicating that an increase in NE concentration is associated with an increase in MEL concentration. MEL plasma concentrations were significantly higher in awake volunteers (anaesthesia type ‘awake’) compared to those under GA (Estimate = 0.36, CI = 0.27–0.44, *p* < 0.001). An interaction between NE and anaesthesia type was explored but not retained in the final model. In this exploratory analysis, the interaction term was not statistically significant (Estimate = −0.02877, *p* = 0.528).

#### 3.3.2 Random Effects

There was significant interindividual variability in intercepts (baseline MEL concentration) for individual volunteers (0.608, CI = 0.379–0.98), suggesting substantial interindividual variability in baseline MEL plasma concentration. The variance in the slope of NE versus MEL was 0.042 (CI = 0.02–0.08), indicating considerable variability in the MEL response to NE administration. A positive correlation (*r* = 0.74) was observed between the random intercept and the slope suggesting that volunteers with higher baseline MEL plasma concentrations tended to exhibit a greater increase in MEL following NE administration.

### 3.4 Sensitivity Analyses

To assess the robustness of the findings to the handling of melatonin concentrations below the LLD, the linear mixed‐effects model was repeated using alternative approaches: assigning values below the LLD to 8 pmol·L^−1^ and excluding these observations. The direction and magnitude of the observed effects were comparable across all analyses. The positive association between norepinephrine and melatonin concentrations (*β* = 0.193–0.230) and the higher melatonin concentrations observed in the awake condition compared with general anaesthesia (*β* = 0.190–0.356) remained statistically significant under all approaches (Supporting Information S2: Table 2).

### 3.5 Model Diagnostics

Four diagnostic plots were generated to validate our model (see Supporting Information S1: Figure 1). The residuals versus fitted values plot showed no major systemic patterns, supporting approximate linearity, although minor deviations were observed at lower fitted values. The normal *Q*–*Q* plot indicated that residuals were approximately normally distributed, with some deviations in the tails. The scale‐location plot suggested broadly homogeneous variance, although slight heteroscedasticity was present. Finally, the Cook's distance did not indicate influential observations.

## 4 Discussion

In this study, the effects of exogenous intravenous NE administration on plasma MEL concentrations in both awake and anaesthetised volunteers were investigated. Intravenous NE administration was associated with an increase in plasma MEL concentrations irrespective of the anaesthesia state. Although there was descriptively lower MEL response to NE during GA, the model did not support the hypothesis that GA modulates this interaction. Interestingly, in four volunteers, NE administration was not associated with a measurable increase in MEL concentration.

Substantial interindividual variability was observed in both baseline MEL plasma concentration and the response to NE administration. Volunteers with a higher baseline MEL concentration exhibited a stronger response to intravenous NE administration. These findings highlight potential inter‐individual physiological differences in the regulation of MEL secretion and metabolism.

### 4.1 Interpretation

Previous studies have investigated the influence of NE on MEL production in vitro [16, 17]. These studies demonstrated that NE interacts with α‐ and β‐adrenoceptors on pinealocytes, triggering intracellular signalling pathway that promotes the conversion of tryptophan to serotonin and finally to MEL [1]. Our study extends this evidence in vivo, showing that intravenous NE administration is associated with higher plasma MEL concentrations.

A previous study in 170 healthy adults showed substantial inter‐individual variability in nighttime salivary MEL concentrations [18], but uniformly low daytime concentrations. This was consistent with reports that plasma MEL concentrations are usually < 10 pg.mL^−1^ during the daytime [19]. Given that our study was conducted during daylight hours, baseline MEL concentrations would be expected to be low. However, concentrations above 10 pmol.L^−1^ were found in 25 patients. Although unexpected, several factors may have contributed to these findings. A delayed circadian phase cannot be excluded, as circadian phase and chronotype were not assessed in this study. Alternatively, increased endogenous catecholamine activity related to anticipatory procedural stress may have stimulated melatonin production.

In this study, substantial inter‐individual variability was observed in the MEL response to intravenous NE administration, with a steeper response in volunteers with higher baseline MEL concentrations. This baseline‐dependent drug response may reflect differences in alpha (and/or) beta adrenoreceptor expression or variability in baseline enzymatic activity. MEL is predominantly metabolised in the liver by CYP1A2, which exhibits marked interindividual variability, with studies reporting up to a ~200‐fold range in mRNA expression and a ~35‐fold range in enzymatic activity [20, 21]. This variability could contribute to differences in both baseline MEL concentrations and individual responsiveness to NE.

MEL concentrations were lower under GA compared to the awake state. This suggests that anaesthesia itself may suppress MEL through mechanisms independent of NE. As no interaction between NE and anaesthesia state was retained in the final model, the effect of NE on MEL appears consistent across all conditions. The lower MEL concentrations under GA may therefore reflect mechanisms independent of NE, such as circadian disruption or direct effect on pinealocyte function.

This aligns with previous findings showing that GA disrupts MEL secretion [11], shifts circadian rhythm phase [6, 22] and contributes to postoperative sleep disturbances [23]. One proposed mechanism involves the enhanced GABAergic activity induced by anaesthetic agents such as propofol, which may inhibit the suprachiasmatic nucleus and directly reduce MEL synthesis at the level of the pineal gland [24, 25].

### 4.2 Implications

MEL secretion is tightly regulated by the circadian system and is normally suppressed in daylight, making it uncommon to observe appreciable plasma MEL concentrations during the day. Our findings suggest that intravenous NE administration increases MEL concentration even in the presence of GA, suggesting that the pinealocytes remain responsive to NE under anaesthesia. Importantly, the MEL concentrations observed in our study remained within the physiological nighttime range (~50–150 pmol.L^−1^) and did not reach supraphysiological levels.

The functional role of MEL in perioperative physiology and critical illness remains incompletely understood. Beyond its role in circadian regulation, MEL has antioxidant, anti‐inflammatory and immunomodulatory properties that may be relevant to the physiological response to surgical injury. In neonates undergoing surgery, perioperative MEL administration reduced markers of oxidative stress and circulating inflammatory mediators, including interleukin‐6, interleukin‐8 and tumour necrosis factor‐α [26]. In an experimental model of hepatic ischaemia–reperfusion injury, MEL also modified neutrophil apoptosis, supporting a potential effect on innate immune responses [27]. Experimental studies have further demonstrated enhanced angiogenesis and wound healing following MEL treatment [28], together with accelerated wound maturation and increased collagen deposition in diabetic wounds [29].

Clinical studies provide some evidence that these biological effects may translate into aspects of postoperative recovery. Perioperative MEL administration has been associated with lower postoperative pain and analgesic requirements and improved sleep quality after prostatectomy [30], as well as improved early quality of recovery after bariatric surgery [31]. Collectively, these studies indicate that MEL can influence inflammatory and immune responses, tissue repair and clinical domains relevant to postoperative recovery.

Most of this evidence concerns pharmacological administration of exogenous MEL and therefore does not establish that the physiological increase in endogenous MEL observed in our study has comparable effects. Nevertheless, the preserved response to NE during GA indicates that adrenergic–pineal signalling remains active under anaesthesia and may contribute to perioperative regulation of endogenous MEL. Human studies showing that β‐adrenoceptor blockade suppresses nocturnal MEL secretion further indicate that this pathway is pharmacologically modifiable [32].

This raises the possibility that the choice of perioperative cardiovascular medication may influence endogenous MEL secretion. For example, future studies could compare antihypertensive agents that inhibit β‐adrenergic signalling (e.g., β‐blockers) with agents acting through non‐adrenergic mechanisms (e.g., Ca‐antagonists), or vasopressors with different adrenergic receptor profiles. (e.g., norepinephrine vs. phenylephrine). Whether such differences affect perioperative MEL concentrations or patient recovery remains unknown and should be evaluated before adrenergic drug selection can be considered for this purpose.

The marked inter‐individual variability in the response to NE raises the possibility that patients differ in their capacity to generate endogenous MEL. Such differences could contribute to heterogeneous responses observed in studies of perioperative MEL supplementation. If confirmed in clinical populations, assessment of endogenous MEL responsiveness might help identify patients more likely to benefit from targeted supplementation or other circadian‐based interventions. Future studies should therefore determine whether inter‐individual differences in the endogenous MEL response are associated with postoperative inflammation, immune function, tissue repair, sleep quality or overall recovery, and whether they modify the effects of perioperative MEL supplementation.

### 4.3 Strengths and Limitations

The main strength of our study is the rigorous study design and study execution, where intravenous NE administration and blood sampling were conducted according to a predefined protocol in healthy volunteers without any co‐medication or surgical intervention. This controlled environment allowed us to precisely investigate the relationship between MEL and NE without confounding factors that could potentially influence the results. By isolating these variables, we could focus solely on the NE‐MEL relationship, providing clear and unambiguous data on this interaction.

However, these stringent conditions also present a limitation, as they do not fully replicate the complexities of clinical settings where multiple factors, such as co‐medications, illness severity and surgical stress, can influence MEL plasma concentration. This controlled environment might limit the generalisability of our findings to broader patient populations in real‐world medical scenarios. Despite this, our primary objective was to elucidate the NE–MEL relationship in a controlled setting, and we believe that this approach was the most appropriate for achieving that goal. Future studies should aim to validate these findings in more varied and clinically relevant conditions to enhance their applicability to patient care.

The imputation of a value of 4 pmol.L^−1^ to samples with MEL concentrations below the LLD could introduce bias. We considered several solutions, such as excluding all these samples or assigning values of 0.1, 4 or 8 pmol.L^−1^. Excluding these samples could introduce bias by omitting potentially informative low MEL concentrations. Assigning extreme values like 0.1 or 8 pmol.L^−1^ might also introduce bias by making it unlikely that most values are accurately approximated. Therefore, we opted to set the value to 4 pmol.L^−1^, which is a method commonly used in studies involving assays [33]. This choice, while not perfect, was intended to provide a more reasonable approximation and maintain the integrity of our data set.

The 30‐min washout period between the awake and general anaesthesia phases was shorter than five multiples of the melatonin half‐life (i.e., 5 × 45 min) which generally considered necessary for near‐complete elimination of a molecule. Therefore, some carry‐over of circulating melatonin cannot be excluded. However, as can be seen in Table 1, the baseline concentrations present before the first (awake) phase of the study were higher than the subsequent concentrations prior to the start of the second (general anaesthesia) phase of the study. Moreover, sensitivity analyses excluding the first two measurements obtained during general anaesthesia yielded similar results (Supporting Information S2: Table 3), suggesting that any potential carry‐over did not substantially influence the observed association between norepinephrine and melatonin.

Finally, general anaesthesia itself may have influenced melatonin secretion through disruption of circadian regulation, including effects on GABAergic and NMDA‐mediated signalling and clock‐gene expression. Current evidence does not demonstrate that propofol consistently suppresses melatonin production in humans, while direct evidence regarding remifentanil remains limited [7, 11]. It is therefore plausible that the propofol–remifentanil anaesthetic regimen contributed to the attenuated melatonin response observed during general anaesthesia. However, the present study was not designed to distinguish the effects of individual anaesthetic agents from the broader effects of general anaesthesia on circadian regulation and melatonin secretion. Future studies should elucidate the independent effects of propofol, remifentanil and other anaesthetic drugs on melatonin secretion and circadian regulation, thereby distinguishing drug‐specific effects from the broader physiological consequences of anaesthesia.

### 4.4 Future

This secondary analysis provides a foundation for further investigation into the physiological and clinical implications of NE‐induced MEL secretion. Given the clear NE‐induced increase in MEL and the observed inter‐individual variability in both baseline MEL levels and response to stimulation, future studies should focus on clarifying the consequences of the NE–MEL interaction in the perioperative setting.

Moreover, future studies should explore differences between exogenous MEL supplementation and endogenous adrenergic stimulation via NE. While NE promotes a physiological, and likely more gradual MEL release, exogenous MEL leads to abrupt, often supraphysiological plasma levels. Understanding this difference could help understand inconsistent findings in previous trials on MEL supplementation.

## 5 Conclusion

This study shows that intravenous administration of NE significantly increases plasma MEL levels in both awake and anaesthetised individuals. While MEL plasma concentrations were significantly lower under general anaesthesia compared to those awake, the data did not support a modulatory effect of anaesthesia on this interaction. Marked inter‐individual variability in baseline MEL and response to NE suggests underlying differences in MEL secretion and metabolism, highlighting the importance of personalised strategies targeting circadian and cognitive outcomes.

## Author Contributions

**M.M. Sahinovic:** analysis of the data, preparation of the manuscript. **I.N. de Keijzer:** study design, conduct of the study, preparation of the manuscript. **M. Stern:** preparation of the manuscript. **K.M. Vermeulen:** analysis of the data, preparation of the manuscript. **M.M.R.F. Struys:** study design, conduct of the study, preparation of the manuscript. **A.R. Absalom:** study design, conduct of the study, preparation of the manuscript. **J.P. van den Berg:** study design, conduct of the study, preparation of the manuscript. **J.J. Vos:** study design, conduct of the study, preparation of the manuscript. J.P. van den Berg and J.J. Vos are joint senior authors.

## Funding

The authors have nothing to report.

## Ethics Statement

Ethical permission for the study was granted by the BEBO (Stichting Beoordeling Ethiek Biomedisch Onderzoek) Medical Ethics Committee (METc Assen; NL76998.056.21).

## Consent

Written informed consent was obtained from all participants.

## Conflicts of Interest

M.M. Sahinovic, I.N. de Keijzer, M. Stern, K.M. Vermeulen, A.R. Absalom, J.P. van den Berg and J.J. Vos: none to declare. M.M.R.F. Struys: no conflicts of interest directly connected to his work. In general, his research group/department received (over the last 3 years) research grants and consultancy fees from Masimo (Irvine, CA, USA), Becton Dickinson (Eysins, Switzerland), Fresenius‐Kabi (Bad Homburg, Germany), Paion (Aachen, Germany), Medcaptain Europe (Andelst, The Netherlands), Eagle Pharmaceuticals Inc. (Woodcliff, NJ, USA), Noven Pharmaceuticals (Jersey City, NJ, USA) and HanaPharm (Seoul, Republic of Korea). He receives royalties on intellectual property from Demed Medical (Sinaai, Belgium) and Ghent University (Ghent, Belgium).

## Supporting information


Supporting File 1

Supporting File 2

## Data Availability

Data are available upon reasonable request from the corresponding author.
