# Supplementary material for: Influence of Intravenous Norepinephrine Administration on Melatonin Plasma Concentration in Awake and Anaesthetised Healthy Adult Volunteers
Source: J Pineal Res. 2026 Sep 30;78(6):e70188. doi: 10.1111/jpi.70188 (PMC13625809; doi:10.1111/jpi.70188)
Supplement: Supplementary file 2 [file JPI-78-e70188-s002.docx]

**Supplementary material:**

| Study phase | Awake Median (IQR), pmol.L^-1^ | GA Median (IQR), pmol.L^-1^ |
| --- | --- | --- |
| Baseline (0) | *13.7 (8.81–24.3)* | *9.14 (4.0–15.3)* |
| 0.08 µg·kg⁻¹·min⁻¹ | *16.5 (8.41–26.6)* | *8.72 (4.0–16.1)* |
| 0.16 µg·kg⁻¹·min⁻¹ | *29.1 (10.4–43.1)* | *11.3 (4.0–37.6)* |
| 0.20 µg·kg⁻¹·min⁻¹ | *31.7 (12.1–57.2)* | *12.0 (4.0–53.5)* |
| Washout | *12.4 (4.0–26.9)* | *12.2 (4.0–31.1* |

**Table 1.** Median plasma melatonin concentrations at comparable study phases in the Awake and General anaesthesia (GA) conditions. Values are presented as median (interquartile range, IQR) in pmol/mL.

| Sensitivity analysis | N | Noradrenaline β (SE) | P | Awake vs GA β (SE) | P |
| --- | --- | --- | --- | --- | --- |
| <8 pmol.L^-1^ → 4 pmol.L^-1^ | 417 | 0.230 (0.042) | 3.06 × 10⁻⁶ | 0.356 (0.044) | 1.72 × 10⁻¹⁴ |
| <8 pmol.L^-1^ → 8 pmol.L^-1^) | 417 | 0.193 (0.038) | 1.06 × 10⁻⁵ | 0.222 (0.038) | 1.42 × 10⁻⁸ |
| <8 pmol.L^-1^ excluded) | 288 | 0.205 (0.045) | 6.43 × 10⁻⁵ | 0.190 (0.055) | 6.39 × 10⁻⁴ |

**Table 2.** Sensitivity analyses evaluating different approaches for handling melatonin concentrations reported as <8 pmol.l^-1^. The table shows the estimated effects of norepinephrine obtained from the linear mixed-effects model under each approach.

| *Analysis* | *N* | *Noradrenaline β (SE)* | *P* | *Awake vs GA β (SE)* | *P* |
| --- | --- | --- | --- | --- | --- |
| *Primary* | *417* | *0.230 (0.042)* | *3.06 × 10⁻⁶* | *0.356 (0.044)* | *1.72 × 10⁻¹⁴* |
| *Excluding AA5 + AA7* | *361* | *0.188 (0.042)* | *6.62 × 10⁻⁵* | *0.263 (0.049)* | *1.43 × 10⁻⁷* |

**Table 3.** Sensitivity analysis assessing potential carry-over effects. The primary linear mixed-effects model was repeated after excluding the first two general anaesthesia measurements (AA5 and AA7). β denotes the estimated fixed-effect coefficient (SE, standard error).
