# Supplementary material for: Influence of Intravenous Norepinephrine Administration on Melatonin Plasma Concentration in Awake and Anaesthetised Healthy Adult Volunteers
Source: J Pineal Res. 2026 Sep 30;78(6):e70188. doi: 10.1111/jpi.70188 (PMC13625809; doi:10.1111/jpi.70188)
Supplement: Supplementary file 3 [file JPI-78-e70188-s001.docx]

**Supplemental Materials**

**Figure 1. Diagnostic plots linear mixed effects model**
